# Predictive modeling of obstructive sleep apnea using pharyngeal magnetic resonance imaging radiomics and clinical data

**DOI:** 10.5664/jcsm.11706

**Published:** 2025-08-01

**Authors:** Yibin Chen, Heng Xiao, Min Huang, Yingying Zheng, Xiaoyu Dong, Guohao Chen

**Affiliations:** ^1^ Department of Otorhinolaryngology Head and Neck Surgery, The First Affiliated Hospital, Fujian Medical University, Fuzhou, China; ^2^ Department of Otorhinolaryngology Head and Neck Surgery, National Regional Medical Center, Binhai Campus of The First Affiliated Hospital, Fujian Medical University, Fuzhou, China; ^3^ Fujian Institute of Otorhinolaryngology, The First Affiliated Hospital, Fujian Medical University, Fuzhou, China

**Keywords:** obstructive sleep apnea, pharyngeal MRI, radiomics, predictive modeling

## Abstract

**Study Objectives:**

This study aims to assess the predictive performance of models combining pharyngeal magnetic resonance imaging radiomics and clinical data for distinguishing severe and nonsevere obstructive sleep apnea.

**Methods:**

A total of 106 patients were included in the study, with 48 patients having an apnea-hypopnea index < 30 events/h and 58 patients having an apnea-hypopnea index ≥ 30 events/h. Radiomics features were extracted from magnetic resonance imaging images. After applying minimum redundancy and maximum relevance and least absolute shrinkage and selection operator with cross-validation for dimensionality reduction, radiomics models were developed using logistic regression, support vector machine, random forest, and gradient boosting machine. Age and body mass index were used as clinical features to construct a combined model with radiomics features. The performance of the models was evaluated using F1 scores and the area under the receiver operating characteristic curve (AUC).

**Results:**

A total of 129 radiomics features were extracted from magnetic resonance imaging images. Following dimensionality reduction and feature selection, 2 radiomics features with significant predictive value were identified. The combined model, incorporating support vector machine (AUC = 0.78, F1 = 0.75), random forest (AUC = 0.78, F1 = 0.74), gradient boosting machine (AUC = 0.79, F1 = 0.75), and logistic regression (AUC = 0.82, F1 = 0.80), demonstrated superior performance compared to models based solely on radiomics features. The radiomics-only models included support vector machine (AUC = 0.76, F1 = 0.72), random forest (AUC = 0.73, F1 = 0.67), gradient boosting machine (AUC = 0.76, F1 = 0.73), and logistic regression (AUC = 0.78, F1 = 0.76). Among the combined models, logistic regression achieved the highest predictive accuracy and classification performance.

**Conclusions:**

The combined model, integrating radiomics features with clinical characteristics, demonstrates a superior ability to distinguish between severe and nonsevere obstructive sleep apnea. This approach offers a noninvasive and effective new perspective for clinical decision-making.

**Citation:**

Chen Y, Xiao H, Huang M, Zheng Y, Dong X, Chen G. Predictive modeling of obstructive sleep apnea using pharyngeal magnetic resonance imaging radiomics and clinical data. *J Clin Sleep Med*. 2025;21(8):1363–1369.

**BRIEF SUMMARY**

**Current Knowledge/Study Rationale:** Polysomnography remains the gold standard for diagnosing obstructive sleep apnea; however, its time-consuming and inconvenient nature limits its widespread clinical application. Integrating pharyngeal magnetic resonance imaging radiomics with clinical data may provide a more efficient alternative for assessing obstructive sleep apnea severity.

**Study Impact:** This study demonstrates that combining pharyngeal magnetic resonance imaging radiomics with clinical data can achieve high predictive accuracy for severe obstructive sleep apnea. This finding supports a potential new perspective for rapidly and conveniently determining the severity of obstructive sleep apnea.

## INTRODUCTION

Obstructive sleep apnea (OSA) is an increasingly common disorder characterized by repeated upper airway obstructions during sleep, leading to intermittent hypoxia, arousals, and fragmented sleep.^1^ OSA is closely associated with various health issues, including daytime sleepiness, cognitive decline, cardiovascular diseases, and metabolic disorders.^2^^,^^3^ Polysomnography (PSG) is considered the gold standard for diagnosing OSA.^4^ However, in many countries, the capacity for conducting laboratory PSG falls short of demand, resulting in long waiting lists, high examination costs, and numerous untreated suspected OSA cases.^5^ Therefore, there is a need to explore new, quick, and reliable methods for assessing the severity of OSA.

Radiomics is an emerging and promising field that provides comprehensive quantification of imaging data through the high-throughput extraction and analysis of a large number of image features.^6^ In recent years, radiomics techniques have been widely applied in the medical field, proving useful in disease diagnosis and treatment outcome prediction.^7^^–^^9^ By analyzing various imaging data, such as magnetic resonance imaging (MRI) and computed tomography, radiomics can offer more comprehensive disease information.^10^

In this study, we selected various machine learning methods for our radiomics models to explore the relationship between MRI-based radiomics and clinical features in assessing the severity of OSA. Our goal is to provide new perspectives and methods for the diagnosis and classification of patients with OSA, thereby improving clinical decision-making and patient outcomes.

## METHODS

### Patient population and study design

This study collected medical imaging data from 106 patients (48 with nonsevere OSA [apnea-hypopnea index (AHI) < 30 events/h] and 58 with severe OSA [AHI ≥ 30 events/h]) in the Department of Otolaryngology-Head and Neck Surgery of the First Affiliated Hospital of Fujian Medical University between October 2022 and November 2023. Using the 3D Slicer software (The Slicer Community, an international open-source collaboration; http://www.slicer.org), we manually delineated regions of interest (ROIs) on each patient’s images. Radiomics features were extracted using the PyRadiomics package (version 3.1.0; https://pyradiomics.readthedocs.io) implemented in Python (Python Software Foundation, Wilmington, Delaware, USA). The FeatureExtractor method from this package was utilized to extract multiple radiomics features from each ROI for each patient.

To reduce overfitting and improve the model’s generalizability, we initially used the minimum redundancy and maximum relevance method for feature selection.^11^ The minimum redundancy and maximum relevance approach optimizes the feature set by selecting features highly correlated with the target variable while minimizing redundancy among them. The minimum redundancy and maximum relevance method helps prioritize the most informative imaging features, ensuring that the model uses only relevant and nonredundant data to enhance predictive performance. The combination of these 2 methods significantly reduced the number of features, with detailed outcomes of the initial and final number of retained features reported in the Results section.

**Figure 1:**
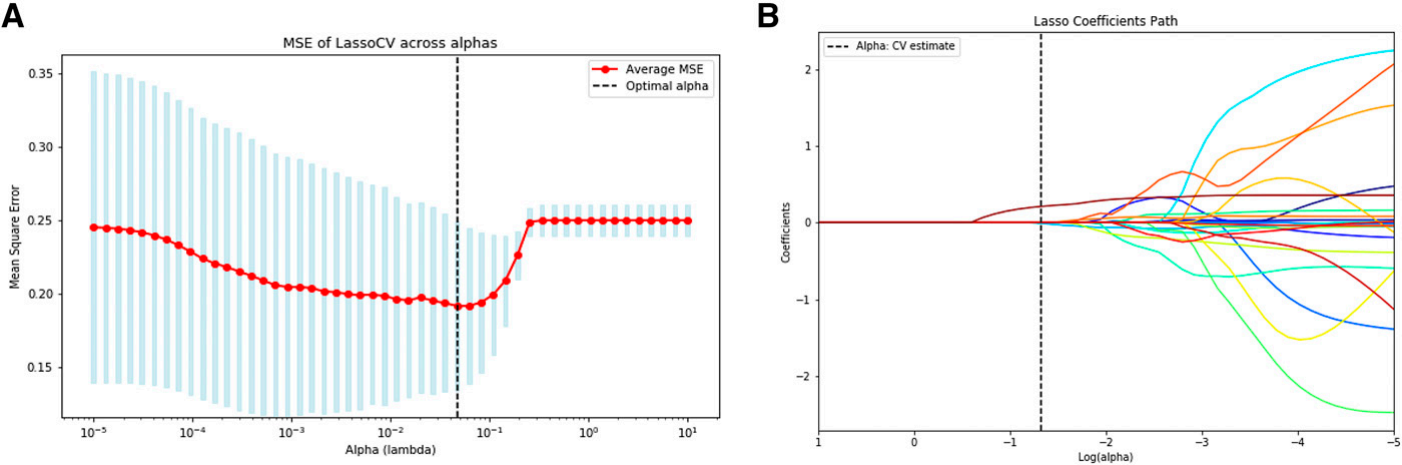
Feature selection using the LASSO regression model. **(A)** The tuning parameter (λ) was determined through 10-fold cross-validation, with the minimum MSE criterion used to select the optimal λ, marked by the vertical dashed line. **(B)** The LASSO coefficient path across different log(λ) values, with the vertical dashed line indicating the optimal λ, showing the nonzero coefficients of selected radiomic features. CV = cross-validation, LASSO = least absolute shrinkage and selection operator, MSE = mean squared error.

Subsequently, we applied least absolute shrinkage and selection operator with cross-validation for further feature selection,^12^ which uses L1 regularization to achieve sparse representation of features (**Figure 1**). Least absolute shrinkage and selection operator with cross-validation automatically eliminates less important features, maintaining a focused set of predictors that improves model accuracy and interpretability. After feature reduction, we trained and predicted using logistic regression (LR), support vector machine (SVM), random forest (RF), and gradient boosting machine (GBM). These machine learning models each provide a different approach to evaluating and predicting patient outcomes, allowing for robust analysis of imaging and clinical data.

To evaluate the models’ stability and generalizability, we employed 5-fold stratified cross-validation. This method splits the dataset into 5 subsets, with 4 subsets used for training and 1 subset for validation in each of the 5 iterations, ensuring that every sample is used for both training and validation. Stratification helps maintain the balance of class distributions, improving the model’s reliability across diverse patient groups.

Age and body mass index (BMI) were incorporated as clinical features to build a combined model alongside the radiomics features. Both radiomics-only models and combined models integrating clinical and radiomics features to differentiate between patients with severe and nonsevere OSA. The models’ performance was assessed using F1 scores and the area under the receiver operating characteristic curve (AUC). Inclusion criteria: (1) patients underwent pharyngeal MRI and PSG; (2) age over 18 years; (3) complete clinical data available. Exclusion criteria: (1) images with severe motion artifacts or significant noise; (2) history of previous pharyngeal surgery; (3) history of pharyngeal tumors; (4) severe cardiovascular or neurological diseases; (5) long-term use of medications that might affect sleep or pharyngeal function. The study was conducted following approval from the medical ethics committee of the First Affiliated Hospital of Fujian Medical University.

### Image acquisition

All patients underwent MRI on a 1.5-T scanner (Skyra, Siemens Healthcare, Erlangen, Germany). The imaging parameters were as follows: echo train length = 14 echoes, repetition time = 3,278.0 ms, echo time = 74.2 ms, and field of view = 26 × 36 cm. The examination focused on the head and neck region, utilizing a T2-weighted fat-suppressed sagittal fast spin echo sequence. The slice thickness was set to 4 mm with an interslice spacing of 4.8 mm.

### Imaging and clinical data analysis

The imaging and clinical data of the patients were sourced from routine clinical records and the Picture Archiving and Communication System at our hospital. We conducted a retrospective analysis, including clinical parameters such as age, sex, height, and weight. All MRI images were evaluated and discussed by 2 experienced radiologists who were blinded to the PSG results and clinical data. To ensure consistency and reliability in the evaluation results, we used the intraclass correlation coefficient to measure interobserver agreement.^13^

### Statistical analyses

Statistical analyses were conducted using IBM SPSS Statistics 25.0 (IBM Corporation, Armonk, New York, USA). Continuous data were expressed as mean ± standard deviation for normally distributed variables or as median (25th percentile, 75th percentile) for nonnormally distributed variables. The Shapiro–Wilk test was used to assess normality. Normally distributed continuous variables were compared using the independent samples *t* test, while nonnormally distributed variables were analyzed using the Mann–Whitney *U* test. Categorical variables were evaluated using the Chi-square test. Correlation analysis between AHI and independent variables was performed using Pearson and Spearman correlation coefficients. A *P* value of < .05 was considered statistically significant.

### Image segmentation

**Figure 2:**
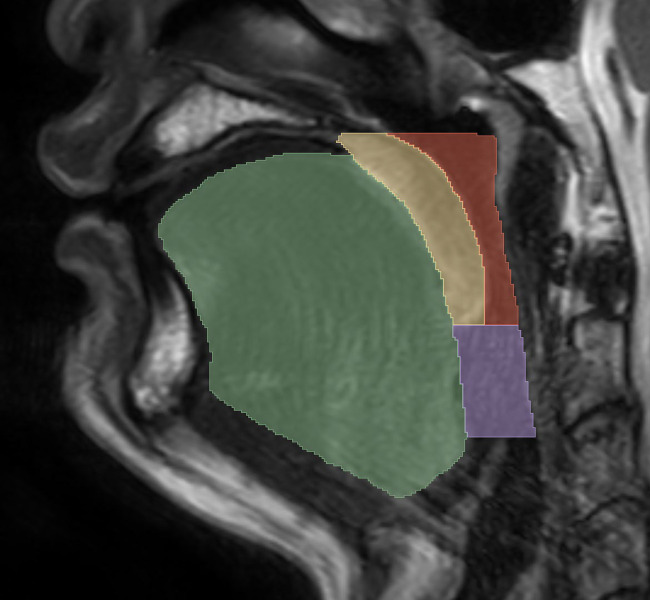
The sagittal MRI image showing the tongue (green), soft palate (yellow), retropalatal airway (red), and retroglossal airway (purple). MRI = magnetic resonance imaging.

All images were saved in Digital Imaging and Communications in Medicine format. Two radiologists, blinded to the patients’ PSG results and clinical data, manually segmented the ROIs using 3D Slicer software (version 5.4.0).^14^ The ROIs included the tongue, soft palate, retroglossal airway, and retropalatal airway. The retropalatal airway was defined as extending from the hard palate to the lower edge of the uvula.^15^^–^^17^ The retroglossal airway was defined as extending from the lower edge of the uvula to the upper edge of the epiglottis.^15^^,^^16^^,^^18^^–^^20^ These definitions are illustrated in the diagram shown in **Figure 2**.

## RESULTS

### Patient imaging and clinical characteristics

**Figure 3:**
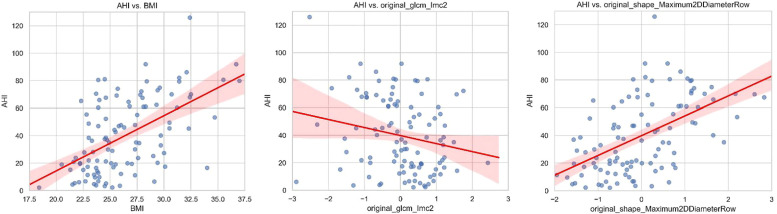
Scatter plots of AHI vs BMI and radiomic features. Scatter plots illustrating AHI vs BMI, original_glcm_Imc2, and original_shape_Maximum2DDiameterRow. The red regression line indicates the linear trend. AHI = apnea-hypopnea index, BMI = body mass index.

In the nonsevere OSA group, 72.9% were male and 27.1% were female, while in the severe OSA group, 87.9% were male and 12.1% were female, with a significant difference in sex distribution between the 2 groups (*P* = .049). The median BMI was 23.85 (25th percentile: 22.63, 75th percentile: 25.80) in the nonsevere OSA group and 27.45 (25th percentile: 24.60, 75th percentile: 30.50) in the severe OSA group, with a statistically significant difference (*P* < .001). The mean age was 37.50 ± 1.55 years in the nonsevere OSA group and 40.52 ± 1.46 years in the severe OSA group, showing no significant difference (*P* = .160). The median AHI was 16.55 events/h (25th percentile: 6.73, 75th percentile: 20.75) in the nonsevere OSA group and 61.05 events/h (25th percentile: 45.35, 75th percentile: 72.38) in the severe OSA group, with a highly significant difference between the 2 groups (*P* < .001). Detailed results are presented in **Table 1**. The correlation analysis demonstrated that BMI and AHI exhibited a moderate positive correlation (Pearson *r* = .5498, *P* < .001; Spearman *r* = .5305, *P* < .001). Similarly, original_shape_Maximum2DDiameterRow and AHI showed a moderate positive correlation (Pearson *r* = .5328, *P* < .001; Spearman *r* = .5530, *P* < .001). In contrast, original_glcm_Imc2 and AHI demonstrated a weak negative correlation (Pearson *r* = −.2181, *P* = .0247; Spearman *r* = −.2502, *P* = .0097). The scatter plots visualizing these correlations are presented in **Figure 3**.

**Table 1:** Demographic and clinical characteristics of the study population.

	Total Population	Nonsevere OSA Group	Severe OSA Group	P*
Sex, n (%)				
Male	86 (81.1)	35 (72.9)	51 (87.9)	.049^a^
Female	20 (18.9)	13 (27.1)	7 (12.1)	
Age (years), mean ± SD	39.15 ± 1.07	37.50 ± 1.55	40.52 ± 1.46	.160^b^
BMI (kg/m²), M (P25, P75)	25.45 (23.70, 28.55)	23.85 (22.63, 25.80)	27.45 (24.60, 30.50)	< .001^c^
AHI (events/h), M (P25, P75)	35.70 (17.23, 62.85)	16.55 (6.73, 20.75)	61.05 (45.35, 72.38)	< .001^c^

^a^Based on Chi-square test. ^b^Based on independent samples *t* test. ^c^Based on Mann–Whitney *U* test. *P values reflect comparisons between the nonsevere OSA group and the severe OSA group. AHI = apnea-hypopnea index, BMI = body mass index, M (P25, P75) = median (25th percentile, 75th percentile), OSA = obstructive sleep apnea, SD = standard deviation.

**Figure 4:**
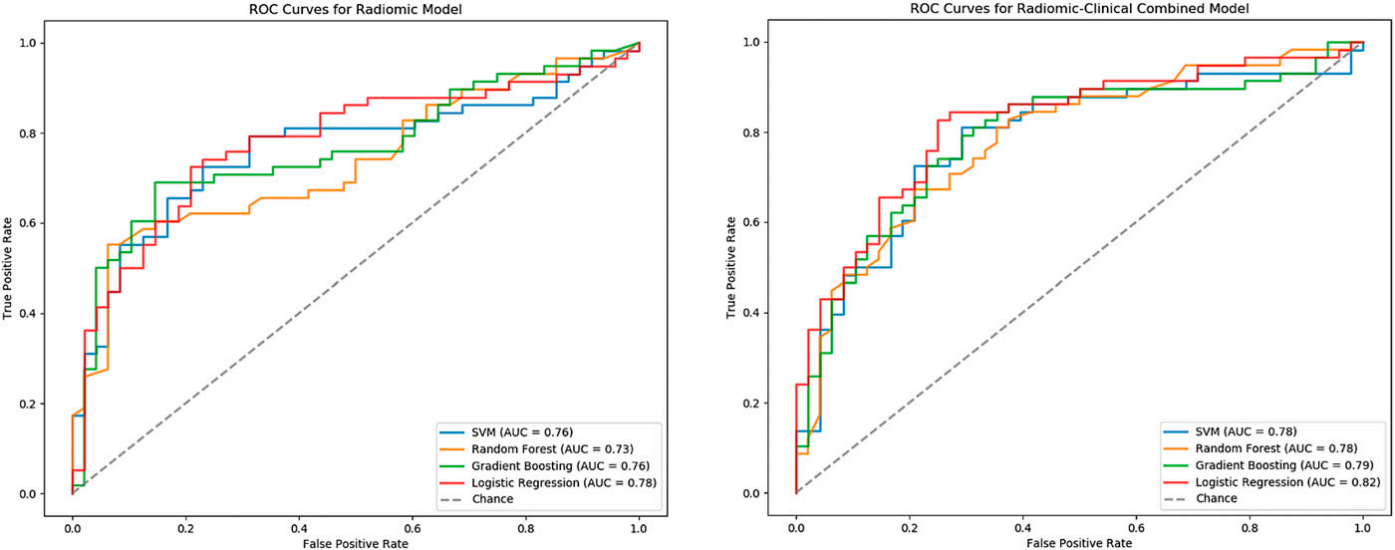
Average AUC for different machine learning methods under radiomics-only and radiomics-clinical combined models. AUC = area under the receiver operating characteristic curve.

A total of 129 radiomics features were extracted from the ROIs, consisting of 7 categories: first-order statistics, shape-based (3D/2D), gray level co-occurrence matrix, gray level run length matrix, gray level size zone matrix, neighboring gray-tone difference matrix, and gray level dependence matrix. The extracted radiomics features demonstrated excellent reproducibility, with an intraclass correlation coefficient greater than .9. After feature reduction and selection, 2 features with nonzero coefficients were retained: original_glcm_Imc2 and original_shape_Maximum2DDiameterRow. original_glcm_Imc2 (informational measure of correlation 2) is a texture-based feature derived from the gray level co-occurrence matrix, which quantifies the complexity of image texture by assessing the spatial relationships between pixel intensities. Original_shape_Maximum2DDiameterRow is a shape-based feature representing the maximum Euclidean distance between 2 points along the row direction in a specific cross-sectional plane of the airway. The combined radiomics-clinical model included 2 additional clinical variables: BMI and age. The receiver operating characteristic curves for the radiomics-only and combined models (including LR, SVM, RF, and GBM) are shown in **Figure 4**.

### Performance of radiomics-only and combined models

For the radiomics-only models, the F1 scores for SVM, RF, GBM, and LR were 0.72, 0.67, 0.73, and 0.76, respectively. For the combined radiomics-clinical models, the F1 scores for SVM, RF, GBM, and LR were 0.75, 0.74, 0.75, and 0.80, respectively.

In terms of AUC scores, the radiomics-only models had AUC values of 0.76, 0.73, 0.76, and 0.78 for SVM, RF, GBM, and LR, respectively, while the combined radiomics-clinical models had AUC values of 0.78, 0.78, 0.79, and 0.82 for SVM, RF, GBM, and LR, respectively. The combined models outperformed the radiomics-only models in both F1 scores and AUC values across all 4 machine learning methods (SVM, RF, GBM, and LR), as shown in **Table 2**.

**Table 2:** Summary of average F1 scores and AUC values for various machine learning methods under radiomics-only and radiomics-clinical combined models.

Machine Learning Method	Radiomic Model F1 Score	Radiomic-Clinical Combined Model F1 Score	Radiomic Model AUC Score	Radiomic-Clinical Combined Model AUC Score
SVM	0.72	0.75	0.76	0.78
RF	0.67	0.74	0.73	0.78
GBM	0.73	0.75	0.76	0.79
LR	0.76	0.80	0.78	0.82

AUC = area under the receiver operating characteristic curve, GBM = gradient boosting machine, LR = logistic regression, RF = random forest, SVM = support vector machine.

## DISCUSSION

This study demonstrates that combining pharyngeal MRI radiomics features with clinical data can effectively distinguish between patients with severe and nonsevere OSA. The LR model, which integrates radiomics features and clinical predictors, achieved satisfactory results in differentiating patients with severe from nonsevere OSA. This approach could provide a convenient and effective method for assessing the severity of OSA, potentially improving clinical decision-making and patient management.

Previous studies have revealed that the incidence of OSA increases with age, and that being overweight or obese is a significant risk factor.^21^ Larger volumes of the tongue and soft palate are potential risk factors for OSA, while a narrowed retrolingual and retropalatal airway also increases the risk of OSA. However, a increased nasopharyngeal airway volume is not considered a major risk factor for OSA.^22^ In this study, we comprehensively considered pharyngeal radiomics features and clinical characteristics. We selected the tongue, soft palate, retrolingual airway, and retropalatal airway from pharyngeal MRI as ROIs for feature extraction and dimensionality reduction. BMI and age were used as clinical features. Using machine learning techniques, we deeply explored the radiomics features of the pharyngeal region and combined this information with clinical data to accurately distinguish between patients with severe and nonsevere OSA.

Radiomics is a noninvasive approach that extracts features from medical images that are not easily assessed by the human eye, providing valuable information to support the diagnosis, prognosis, and treatment of diseases.^6^ By leveraging advanced image analysis techniques, radiomics can extract additional valuable information from routine medical images.^23^ For example, by quantifying imaging features, we can more accurately analyze changes in airway structures and surrounding soft tissues, offering supplementary information on the severity of OSA. In our study, we identified 2 radiomics features, original_glcm_Imc2 and original_shape_Maximum2DDiameterRow, which have significant predictive value. These features may be closely related to specific pathological characteristics of patients with OSA. The feature original_glcm_Imc2 (informational measure of correlation 2) reflects the complexity of image texture, capturing differences in tissue composition and organization in the pharyngeal region. As a texture-based feature derived from the gray level co-occurrence matrix, informational measure of correlation 2 quantifies the spatial relationships between pixel intensities, with higher values indicating greater heterogeneity. In patients with OSA, variations in informational measure of correlation 2 values may reflect structural changes such as fat deposition, muscle tone alterations, or soft tissue remodeling. The feature original_shape_Maximum2DDiameterRow quantifies the maximum Euclidean distance between 2 points along the row direction in a specific cross-sectional plane of the airway, reflecting the widest lateral dimension of the airway within the analyzed MRI slice. This feature provides an objective measurement of airway morphology, offering quantitative insights into the lateral airway space. In the context of OSA, a smaller Maximum2DDiameterRow may indicate greater lateral constriction of the airway, contributing to airway collapsibility during sleep. Compared to patients with nonsevere OSA, those with severe OSA are more likely to exhibit a narrower airway diameter, highlighting this feature as a structural indicator of airway obstruction risk. It can be inferred that there are differences in the texture and shape characteristics of the pharyngeal region between patients with severe and nonsevere OSA.

Research indicates that larger soft tissues and smaller craniofacial dimensions, as measured by MRI, are associated with an increased risk of OSA.^24^^–^^26^ Traditionally, researchers have used multiple regression analysis on these MRI-derived measurements to predict AHI or OSA severity, finding that variables such as tongue volume, soft palate size, and parapharyngeal fat pad volume have predictive value for AHI.^22^ However, due to the complex relationships among these anatomical variables, simple linear models struggle to capture the multifactorial characteristics of OSA development. Consequently, machine learning methods have been increasingly adopted to enhance predictive performance.^27^^,^^28^ Beyond AHI prediction, machine learning has also been widely applied in diagnosing OSA.^29^^–^^32^ In recent years, multiple studies have utilized MRI-derived multidimensional anatomical features combined with machine learning algorithms to improve AHI prediction. For example, researchers have developed deep learning segmentation algorithms to automatically measure upper airway structure volumes and tongue fat content from MRI images, extracting objective indicators for OSA risk assessment.^28^^,^^33^ Bommineni et al employed convolutional neural networks to automatically segment 10 anatomical regions in MRI images, including the tongue, soft palate, and pharyngeal walls, and computed features such as the tongue fat ratio.^34^ Furthermore, radiomics, which extracts advanced imaging features and integrates machine learning algorithms, offers an innovative approach to OSA risk prediction.^28^

This study aims to develop a predictive model for OSA through radiomics analysis of selected ROI in pharyngeal MRI images. Our findings indicate that the combined model, which integrates clinical features, shows an improvement in AUC performance compared to radiomics-only models. This enhancement demonstrates the potential of combining radiomics with clinical information in the diagnostic evaluation of diseases.

In the radiomics-only model, LR exhibited the highest AUC value, indicating its advantage in handling datasets with linear relationships. LR optimizes the classifier by fitting a linear decision boundary, making it particularly suitable for scenarios where the relationships between features are relatively linear and the data contains minimal noise.^35^ SVM, RF, and GBM demonstrated slightly lower performance. This difference in performance may be attributed to the tendency of these models to overfit on small datasets.^36^ Although RF and GBM have strong nonlinear fitting capabilities, they may capture noise in smaller datasets, which can affect the model’s generalizability.^37^^,^^38^ SVM optimizes the classifier by maximizing the margin of the decision boundary, making it excel in handling datasets with complex decision boundaries. However, SVM may face challenges with small datasets. These models typically require more data to be fully trained to avoid overfitting, and their performance may be limited when data are insufficient.^39^ The combined model, which incorporates clinical features, showed improvements in both AUC and F1 scores across all 4 machine learning algorithms. This result highlights the important role of clinical features in enhancing model predictive performance. Among the models, LR performed the best when clinical and radiomics features were combined, likely due to its strong ability to handle linear relationships. This suggests that there may be a linear relationship between the features in the data and the target variable.

Overall, this study demonstrates the feasibility and reliability of combining clinical predictors and radiomics features using LR for comprehensive modeling. This approach offers a promising direction for developing rapid and accurate diagnostic tools for assessing OSA severity.

However, this study has some limitations. First, it is a retrospective, single-center study, which may introduce potential selection bias. Further prospective studies are needed to investigate and validate whether radiomics can enhance model performance. Second, there is some subjectivity in manual segmentation, which may lead to variability. We hope to achieve full automation through deep learning in the future. Third, this study only classified OSA into severe and nonsevere categories. Future research should further validate this approach across different OSA severity levels.

## DISCLOSURE STATEMENT

All authors have reviewed and approved the final version of this manuscript for submission. This work was supported by The 2022 Special Fiscal Fund (BPB-2022CGH). The authors report no conflicts of interest.

## ABBREVIATIONS

AHI: apnea-hypopnea index
AUC: area under the receiver operating characteristic curve
BMI: body mass index
GBM: gradient boosting machine
LR: logistic regression
MRI: magnetic resonance imaging
OSA: obstructive sleep apnea
PSG: polysomnography
RF: random forest
ROI: region of interest
SVM: support vector machine

## OPEN ACCESS

Copyright 2025 The Authors. This is an open access article, distributed under the Creative Commons Attribution 4.0 International License. Sharing and adaptation are permitted provided attribution to its original publication in the Journal of Clinical Sleep Medicine is made in accordance with the license.
